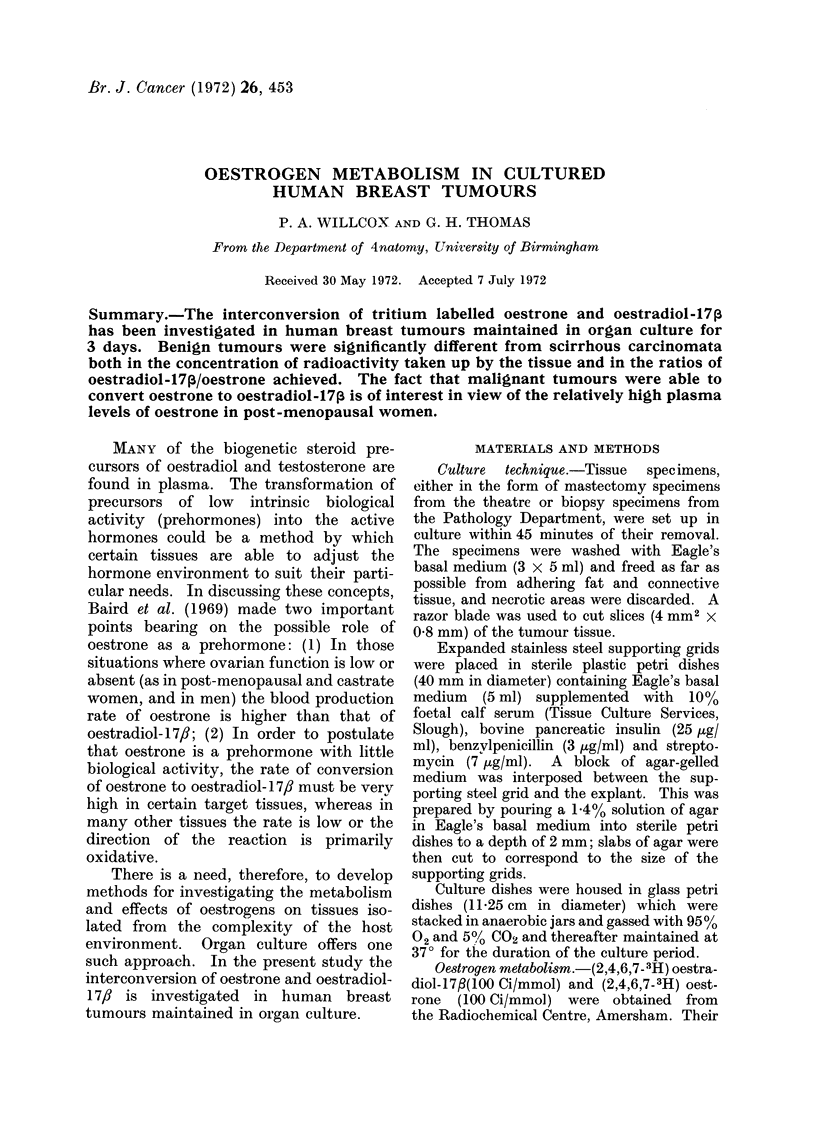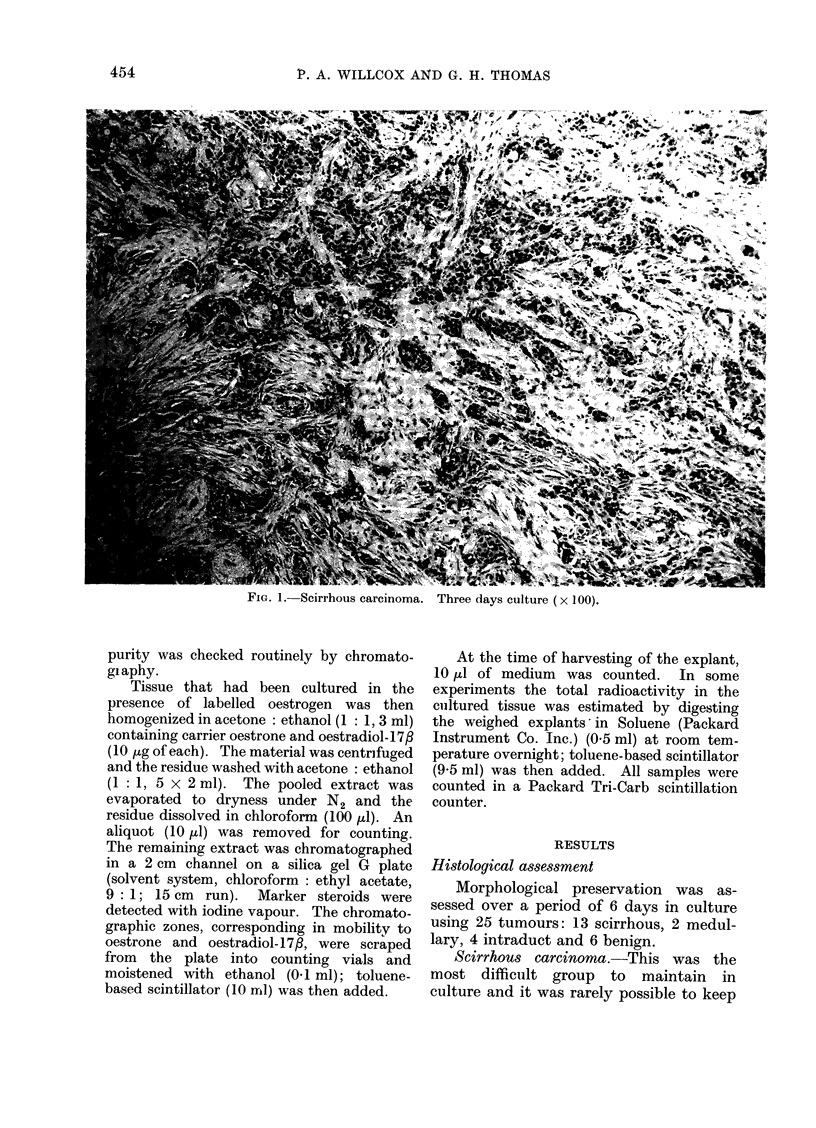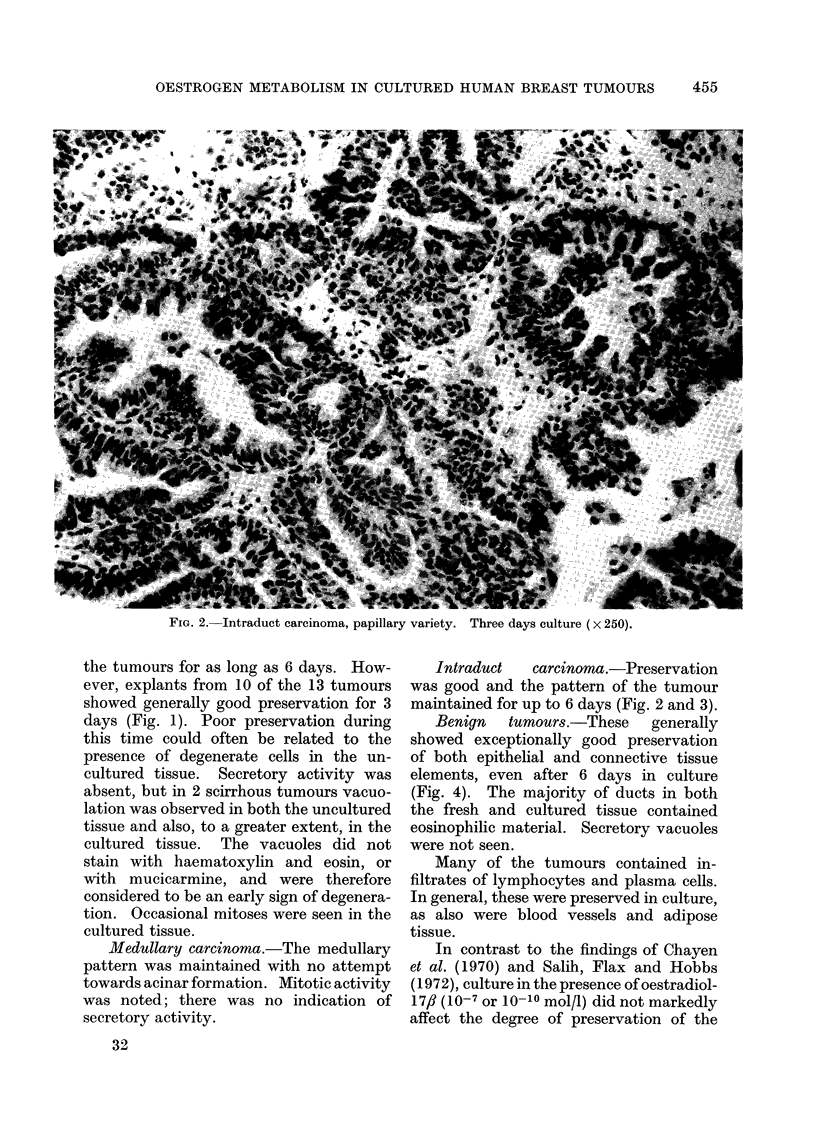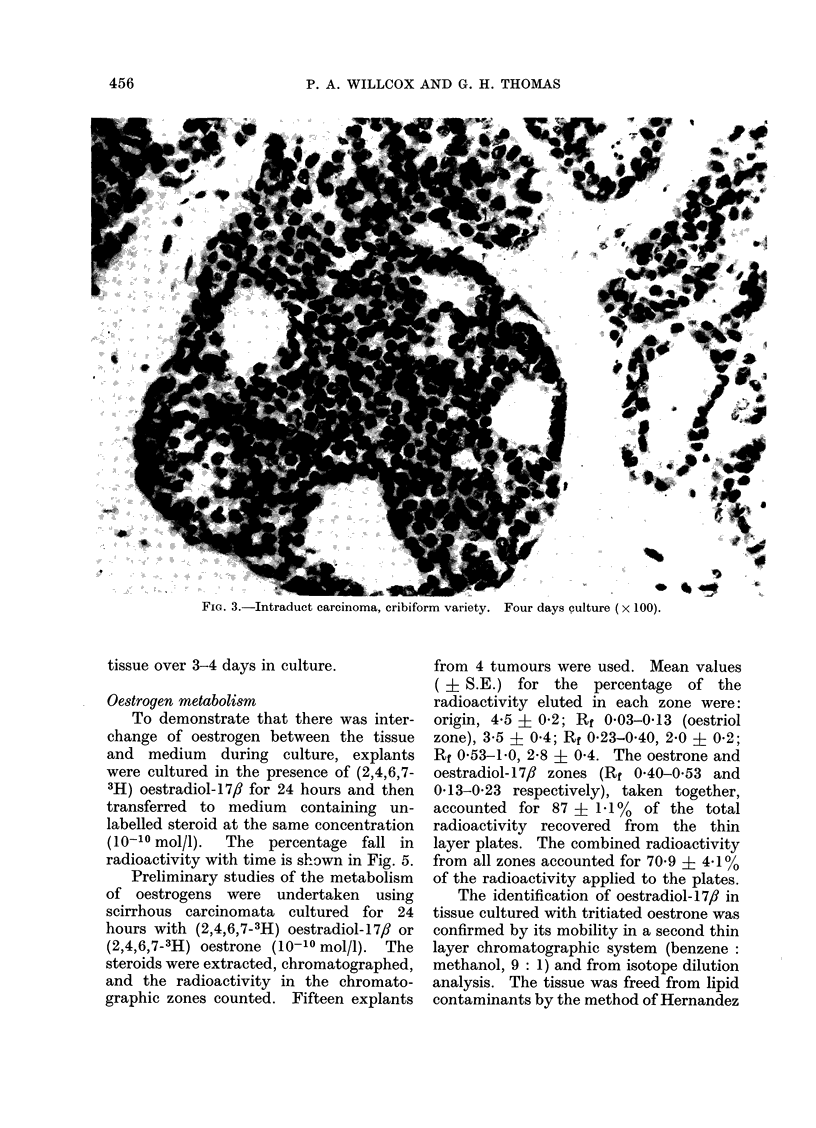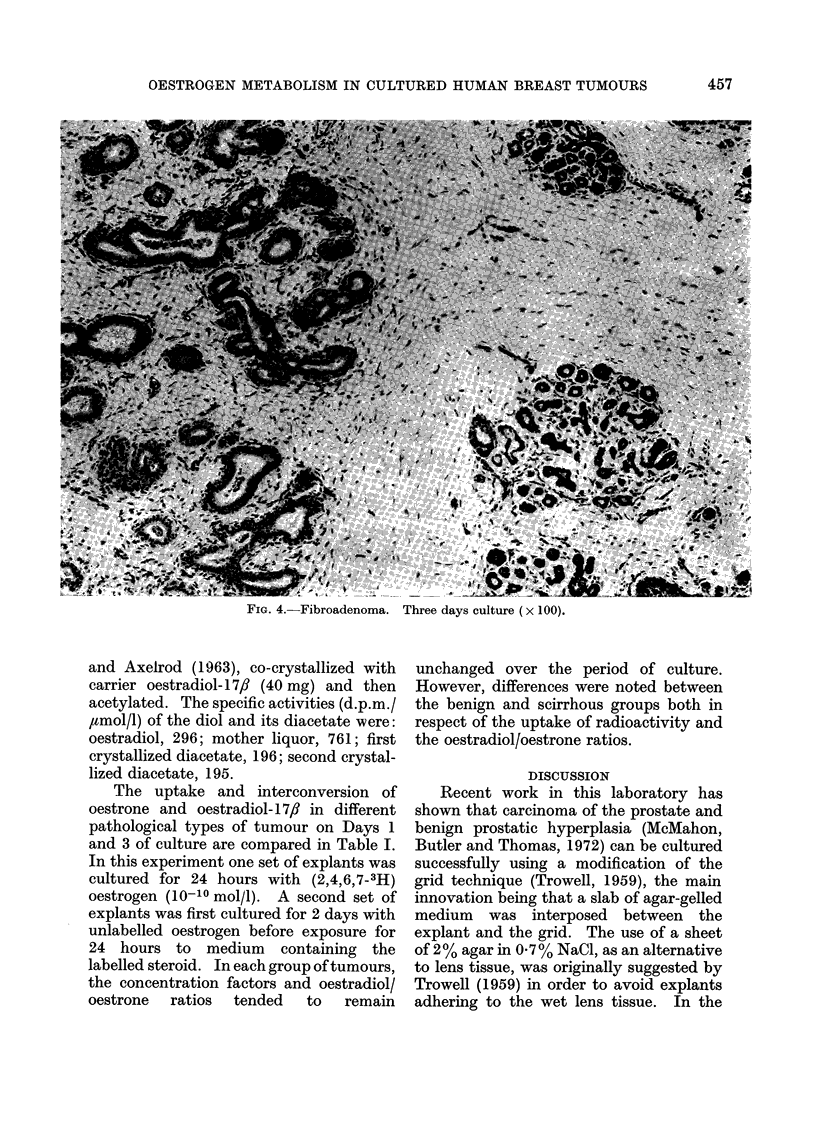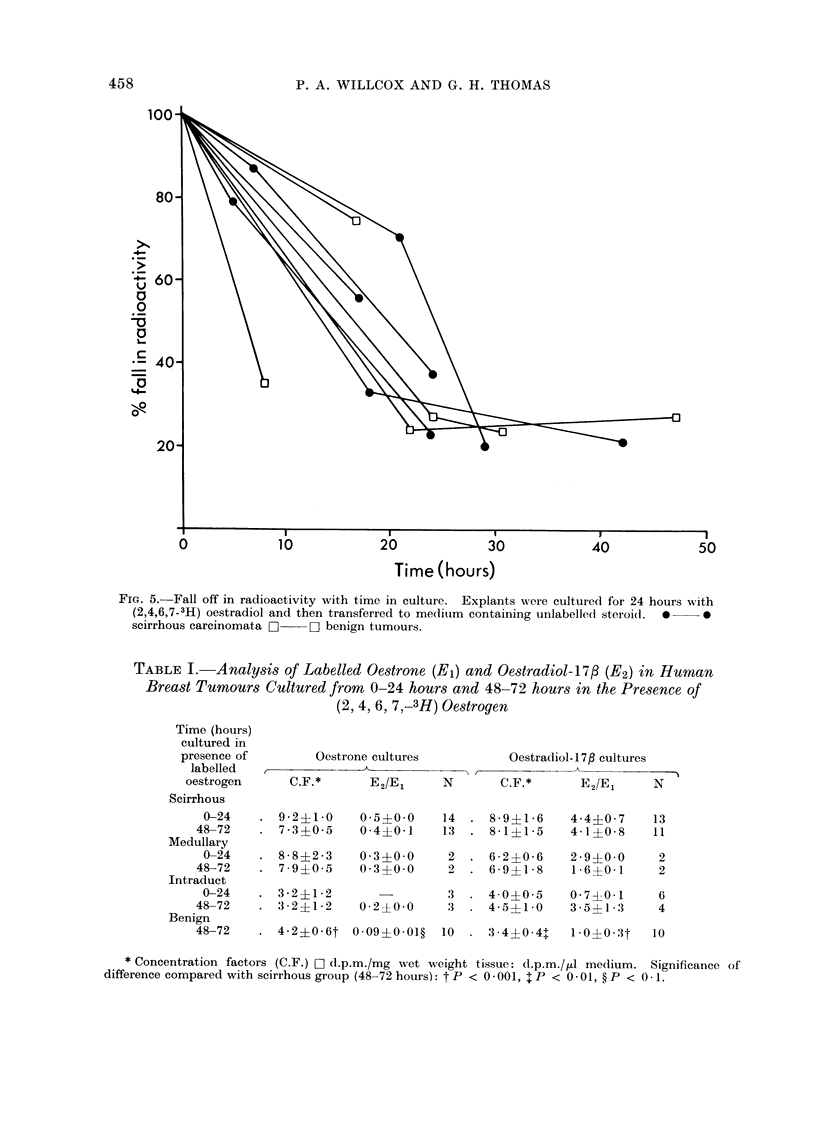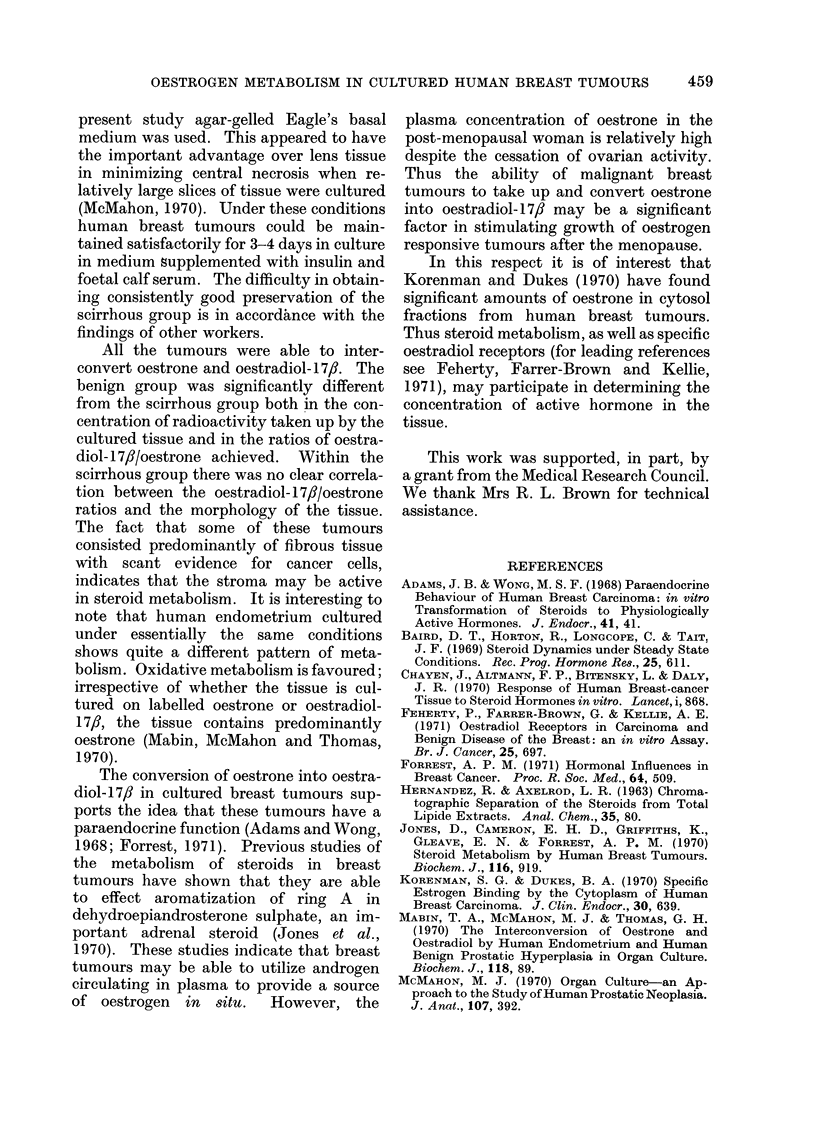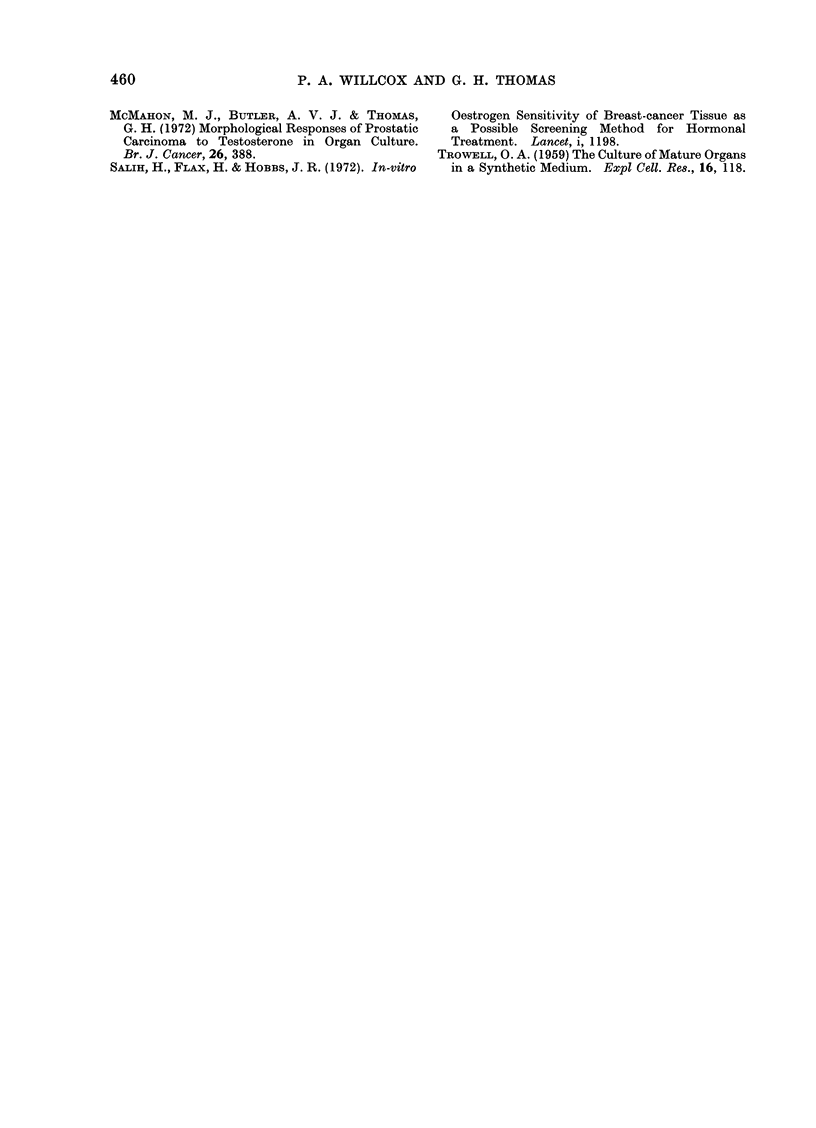# Oestrogen Metabolism in Cultured Human Breast Tumours

**DOI:** 10.1038/bjc.1972.62

**Published:** 1972-12

**Authors:** P. A. Willcox, G. H. Thomas

## Abstract

**Images:**


*Br. J. Cancer* (1972) **26**, 453

### OESTROGEN METABOLISM IN CULTURED HUMAN BREAST TUMOURS

P. A. WILLCOX AND G. H. THOMAS

*From the Department of Anatomy, University of Birmingham*

Received 30 May 1972. Accepted 7 July 1972

**Summary.—The interconversion of tritium labelled oestrone and oestradiol-17β has been investigated in human breast tumours maintained in organ culture for 3 days. Benign tumours were significantly different from scirrhous carcinomata both in the concentration of radioactivity taken up by the tissue and in the ratios of oestradiol-17β/oestrone achieved. The fact that malignant tumours were able to convert oestrone to oestradiol-17β is of interest in view of the relatively high plasma levels of oestrone in post-menopausal women.**

MANY of the biogenetic steroid precursors of oestradiol and testosterone are found in plasma. The transformation of precursors of low intrinsic biological activity (prehormones) into the active hormones could be a method by which certain tissues are able to adjust the hormone environment to suit their particular needs. In discussing these concepts, Baird *et al.* (1969) made two important points bearing on the possible role of oestrone as a prehormone: (1) In those situations where ovarian function is low or absent (as in post-menopausal and castrate women, and in men) the blood production rate of oestrone is higher than that of oestradiol-17$\beta$; (2) In order to postulate that oestrone is a prehormone with little biological activity, the rate of conversion of oestrone to oestradiol-17$\beta$ must be very high in certain target tissues, whereas in many other tissues the rate is low or the direction of the reaction is primarily oxidative.

There is a need, therefore, to develop methods for investigating the metabolism and effects of oestrogens on tissues isolated from the complexity of the host environment. Organ culture offers one such approach. In the present study the interconversion of oestrone and oestradiol-17$\beta$ is investigated in human breast tumours maintained in organ culture.

### MATERIALS AND METHODS

*Culture technique.*—Tissue specimens, either in the form of mastectomy specimens from the theatre or biopsy specimens from the Pathology Department, were set up in culture within 45 minutes of their removal. The specimens were washed with Eagle's basal medium (3 $\times$ 5 ml) and freed as far as possible from adhering fat and connective tissue, and necrotic areas were discarded. A razor blade was used to cut slices (4 mm$^2$ $\times$ 0·8 mm) of the tumour tissue.

Expanded stainless steel supporting grids were placed in sterile plastic petri dishes (40 mm in diameter) containing Eagle's basal medium (5 ml) supplemented with 10% foetal calf serum (Tissue Culture Services, Slough), bovine pancreatic insulin (25 $\mu$g/ml), benzylpenicillin (3 $\mu$g/ml) and streptomycin (7 $\mu$g/ml). A block of agar-gelled medium was interposed between the supporting steel grid and the explant. This was prepared by pouring a 1·4% solution of agar in Eagle's basal medium into sterile petri dishes to a depth of 2 mm; slabs of agar were then cut to correspond to the size of the supporting grids.

Culture dishes were housed in glass petri dishes (11·25 cm in diameter) which were stacked in anaerobic jars and gassed with 95% $O_2$ and 5% $CO_2$ and thereafter maintained at 37° for the duration of the culture period.

*Oestrogen metabolism.*—(2,4,6,7-$^3$H) oestradiol-17$\beta$(100 Ci/mmol) and (2,4,6,7-$^3$H) oestrone (100 Ci/mmol) were obtained from the Radiochemical Centre, Amersham. Their

FIG. 1.—Scirrhous carcinoma. Three days culture (× 100).

purity was checked routinely by chromatography.

Tissue that had been cultured in the presence of labelled oestrogen was then homogenized in acetone : ethanol (1 : 1, 3 ml) containing carrier oestrone and oestradiol-17$\beta$ (10 $\mu$g of each). The material was centrifuged and the residue washed with acetone : ethanol (1 : 1, 5 × 2 ml). The pooled extract was evaporated to dryness under $N_2$ and the residue dissolved in chloroform (100 $\mu$l). An aliquot (10 $\mu$l) was removed for counting. The remaining extract was chromatographed in a 2 cm channel on a silica gel G plate (solvent system, chloroform : ethyl acetate, 9 : 1; 15 cm run). Marker steroids were detected with iodine vapour. The chromatographic zones, corresponding in mobility to oestrone and oestradiol-17$\beta$, were scraped from the plate into counting vials and moistened with ethanol (0·1 ml); toluene-based scintillator (10 ml) was then added.

At the time of harvesting of the explant, 10 $\mu$l of medium was counted. In some experiments the total radioactivity in the cultured tissue was estimated by digesting the weighed explants in Soluene (Packard Instrument Co. Inc.) (0·5 ml) at room temperature overnight; toluene-based scintillator (9·5 ml) was then added. All samples were counted in a Packard Tri-Carb scintillation counter.

### RESULTS

### *Histological assessment*

Morphological preservation was assessed over a period of 6 days in culture using 25 tumours: 13 scirrhous, 2 medullary, 4 intraduct and 6 benign.

*Scirrhous carcinoma.*—This was the most difficult group to maintain in culture and it was rarely possible to keep

FIG. 2.—Intraduct carcinoma, papillary variety. Three days culture (×250).

the tumours for as long as 6 days. However, explants from 10 of the 13 tumours showed generally good preservation for 3 days (Fig. 1). Poor preservation during this time could often be related to the presence of degenerate cells in the uncultured tissue. Secretory activity was absent, but in 2 scirrhous tumours vacuolation was observed in both the uncultured tissue and also, to a greater extent, in the cultured tissue. The vacuoles did not stain with haematoxylin and eosin, or with mucicarmine, and were therefore considered to be an early sign of degeneration. Occasional mitoses were seen in the cultured tissue.

*Medullary carcinoma.*—The medullary pattern was maintained with no attempt towards acinar formation. Mitotic activity was noted; there was no indication of secretory activity.

*Intraduct carcinoma.*—Preservation was good and the pattern of the tumour maintained for up to 6 days (Fig. 2 and 3).

*Benign tumours.*—These generally showed exceptionally good preservation of both epithelial and connective tissue elements, even after 6 days in culture (Fig. 4). The majority of ducts in both the fresh and cultured tissue contained eosinophilic material. Secretory vacuoles were not seen.

Many of the tumours contained infiltrates of lymphocytes and plasma cells. In general, these were preserved in culture, as also were blood vessels and adipose tissue.

In contrast to the findings of Chayen *et al.* (1970) and Salih, Flax and Hobbs (1972), culture in the presence of oestradiol-17$\beta$ ($10^{-7}$ or $10^{-10}$ mol/l) did not markedly affect the degree of preservation of the

FIG. 3.—Intraduct carcinoma, cribiform variety. Four days culture ($\times$ 100).

tissue over 3–4 days in culture.

*Oestrogen metabolism*

To demonstrate that there was interchange of oestrogen between the tissue and medium during culture, explants were cultured in the presence of (2,4,6,7-$^3$H) oestradiol-17$\beta$ for 24 hours and then transferred to medium containing unlabelled steroid at the same concentration ($10^{-10}$ mol/l). The percentage fall in radioactivity with time is shown in Fig. 5.

Preliminary studies of the metabolism of oestrogens were undertaken using scirrhous carcinomata cultured for 24 hours with (2,4,6,7-$^3$H) oestradiol-17$\beta$ or (2,4,6,7-$^3$H) oestrone ($10^{-10}$ mol/l). The steroids were extracted, chromatographed, and the radioactivity in the chromatographic zones counted. Fifteen explants from 4 tumours were used. Mean values ($\pm$ S.E.) for the percentage of the radioactivity eluted in each zone were: origin, 4·5 $\pm$ 0·2; $R_f$ 0·03–0·13 (oestriol zone), 3·5 $\pm$ 0·4; $R_f$ 0·23–0·40, 2·0 $\pm$ 0·2; $R_f$ 0·53–1·0, 2·8 $\pm$ 0·4. The oestrone and oestradiol-17$\beta$ zones ($R_f$ 0·40–0·53 and 0·13–0·23 respectively), taken together, accounted for 87 $\pm$ 1·1% of the total radioactivity recovered from the thin layer plates. The combined radioactivity from all zones accounted for 70·9 $\pm$ 4·1% of the radioactivity applied to the plates.

The identification of oestradiol-17$\beta$ in tissue cultured with tritiated oestrone was confirmed by its mobility in a second thin layer chromatographic system (benzene : methanol, 9 : 1) and from isotope dilution analysis. The tissue was freed from lipid contaminants by the method of Hernandez

FIG. 4.—Fibroadenoma. Three days culture ($\times 100$).

and Axelrod (1963), co-crystallized with carrier oestradiol-17$\beta$ (40 mg) and then acetylated. The specific activities (d.p.m./$\mu$mol/l) of the diol and its diacetate were: oestradiol, 296; mother liquor, 761; first crystallized diacetate, 196; second crystallized diacetate, 195.

The uptake and interconversion of oestrone and oestradiol-17$\beta$ in different pathological types of tumour on Days 1 and 3 of culture are compared in Table I. In this experiment one set of explants was cultured for 24 hours with (2,4,6,7-$^3$H) oestrogen ($10^{-10}$ mol/l). A second set of explants was first cultured for 2 days with unlabelled oestrogen before exposure for 24 hours to medium containing the labelled steroid. In each group of tumours, the concentration factors and oestradiol/oestrone ratios tended to remain unchanged over the period of culture. However, differences were noted between the benign and scirrhous groups both in respect of the uptake of radioactivity and the oestradiol/oestrone ratios.

### DISCUSSION

Recent work in this laboratory has shown that carcinoma of the prostate and benign prostatic hyperplasia (McMahon, Butler and Thomas, 1972) can be cultured successfully using a modification of the grid technique (Trowell, 1959), the main innovation being that a slab of agar-gelled medium was interposed between the explant and the grid. The use of a sheet of 2% agar in 0·7% NaCl, as an alternative to lens tissue, was originally suggested by Trowell (1959) in order to avoid explants adhering to the wet lens tissue. In the

FIG. 5.—Fall off in radioactivity with time in culture. Explants were cultured for 24 hours with (2,4,6,7-$^3$H) oestradiol and then transferred to medium containing unlabelled steroid. ● —— ● scirrhous carcinomata □ —— □ benign tumours.

TABLE I.—*Analysis of Labelled Oestrone* ($E_1$) *and Oestradiol*-17β ($E_2$) *in Human Breast Tumours Cultured from* 0–24 *hours and* 48–72 *hours in the Presence of* (2, 4, 6, 7,–$^3$*H*) *Oestrogen*

| Time (hours) cultured in presence of labelled oestrogen | Oestrone cultures | | | Oestradiol-17β cultures | | |
|---|---|---|---|---|---|---|
| | C.F.* | $E_2/E_1$ | N | C.F.* | $E_2/E_1$ | N |
| Scirrhous | | | | | | |
| 0–24 | 9·2±1·0 | 0·5±0·0 | 14 | 8·9±1·6 | 4·4±0·7 | 13 |
| 48–72 | 7·3±0·5 | 0·4±0·1 | 13 | 8·1±1·5 | 4·1±0·8 | 11 |
| Medullary | | | | | | |
| 0–24 | 8·8±2·3 | 0·3±0·0 | 2 | 6·2±0·6 | 2·9±0·0 | 2 |
| 48–72 | 7·9±0·5 | 0·3±0·0 | 2 | 6·9±1·8 | 1·6±0·1 | 2 |
| Intraduct | | | | | | |
| 0–24 | 3·2±1·2 | — | 3 | 4·0±0·5 | 0·7±0·1 | 6 |
| 48–72 | 3·2±1·2 | 0·2±0·0 | 3 | 4·5±1·0 | 3·5±1·3 | 4 |
| Benign | | | | | | |
| 48–72 | 4·2±0·6† | 0·09±0·01§ | 10 | 3·4±0·4‡ | 1·0±0·3† | 10 |

* Concentration factors (C.F.) □ d.p.m./mg wet weight tissue: d.p.m./μl medium. Significance of difference compared with scirrhous group (48–72 hours): † $P < 0{\cdot}001$, ‡ $P < 0{\cdot}01$, § $P < 0{\cdot}1$.

present study agar-gelled Eagle's basal medium was used. This appeared to have the important advantage over lens tissue in minimizing central necrosis when relatively large slices of tissue were cultured (McMahon, 1970). Under these conditions human breast tumours could be maintained satisfactorily for 3–4 days in culture in medium supplemented with insulin and foetal calf serum. The difficulty in obtaining consistently good preservation of the scirrhous group is in accordance with the findings of other workers.

All the tumours were able to interconvert oestrone and oestradiol-17$\beta$. The benign group was significantly different from the scirrhous group both in the concentration of radioactivity taken up by the cultured tissue and in the ratios of oestradiol-17$\beta$/oestrone achieved. Within the scirrhous group there was no clear correlation between the oestradiol-17$\beta$/oestrone ratios and the morphology of the tissue. The fact that some of these tumours consisted predominantly of fibrous tissue with scant evidence for cancer cells, indicates that the stroma may be active in steroid metabolism. It is interesting to note that human endometrium cultured under essentially the same conditions shows quite a different pattern of metabolism. Oxidative metabolism is favoured; irrespective of whether the tissue is cultured on labelled oestrone or oestradiol-17$\beta$, the tissue contains predominantly oestrone (Mabin, McMahon and Thomas, 1970).

The conversion of oestrone into oestradiol-17$\beta$ in cultured breast tumours supports the idea that these tumours have a paraendocrine function (Adams and Wong, 1968; Forrest, 1971). Previous studies of the metabolism of steroids in breast tumours have shown that they are able to effect aromatization of ring A in dehydroepiandrosterone sulphate, an important adrenal steroid (Jones *et al.*, 1970). These studies indicate that breast tumours may be able to utilize androgen circulating in plasma to provide a source of oestrogen *in situ*. However, the plasma concentration of oestrone in the post-menopausal woman is relatively high despite the cessation of ovarian activity. Thus the ability of malignant breast tumours to take up and convert oestrone into oestradiol-17$\beta$ may be a significant factor in stimulating growth of oestrogen responsive tumours after the menopause.

In this respect it is of interest that Korenman and Dukes (1970) have found significant amounts of oestrone in cytosol fractions from human breast tumours. Thus steroid metabolism, as well as specific oestradiol receptors (for leading references see Feherty, Farrer-Brown and Kellie, 1971), may participate in determining the concentration of active hormone in the tissue.

This work was supported, in part, by a grant from the Medical Research Council. We thank Mrs R. L. Brown for technical assistance.

### REFERENCES

Adams, J. B. & Wong, M. S. F. (1968) Paraendocrine Behaviour of Human Breast Carcinoma: *in vitro* Transformation of Steroids to Physiologically Active Hormones. *J. Endocr.*, **41**, 41.

Baird, D. T., Horton, R., Longcope, C. & Tait, J. F. (1969) Steroid Dynamics under Steady State Conditions. *Rec. Prog. Hormone Res.*, **25**, 611.

Chayen, J., Altmann, F. P., Bitensky, L. & Daly, J. R. (1970) Response of Human Breast-cancer Tissue to Steroid Hormones *in vitro*. *Lancet*, i, 868.

Feherty, P., Farrer-Brown, G. & Kellie, A. E. (1971) Oestradiol Receptors in Carcinoma and Benign Disease of the Breast: an *in vitro* Assay. *Br. J. Cancer*, **25**, 697.

Forrest, A. P. M. (1971) Hormonal Influences in Breast Cancer. *Proc. R. Soc. Med.*, **64**, 509.

Hernandez, R. & Axelrod, L. R. (1963) Chromatographic Separation of the Steroids from Total Lipide Extracts. *Anal. Chem.*, **35**, 80.

Jones, D., Cameron, E. H. D., Griffiths, K., Gleave, E. N. & Forrest, A. P. M. (1970) Steroid Metabolism by Human Breast Tumours. *Biochem. J.*, **116**, 919.

Korenman, S. G. & Dukes, B. A. (1970) Specific Estrogen Binding by the Cytoplasm of Human Breast Carcinoma. *J. Clin. Endocr.*, **30**, 639.

Mabin, T. A., McMahon, M. J. & Thomas, G. H. (1970) The Interconversion of Oestrone and Oestradiol by Human Endometrium and Human Benign Prostatic Hyperplasia in Organ Culture. *Biochem. J.*, **118**, 89.

McMahon, M. J. (1970) Organ Culture—an Approach to the Study of Human Prostatic Neoplasia. *J. Anat.*, **107**, 392.

McMahon, M. J., Butler, A. V. J. & Thomas, G. H. (1972) Morphological Responses of Prostatic Carcinoma to Testosterone in Organ Culture. *Br. J. Cancer*, **26**, 388.

Salih, H., Flax, H. & Hobbs, J. R. (1972). *In-vitro* Oestrogen Sensitivity of Breast-cancer Tissue as a Possible Screening Method for Hormonal Treatment. *Lancet*, i, 1198.

Trowell, O. A. (1959) The Culture of Mature Organs in a Synthetic Medium. *Expl Cell. Res.*, **16**, 118.